# Letter to the Editor on “Atrial fibrillation prevalence and risk profile from novel community-based screening in Thailand: A prospective multi-centre study”

**DOI:** 10.1016/j.ijcha.2021.100733

**Published:** 2021-02-22

**Authors:** Ming-Liang Zuo, Chun-Ka Wong, Lixue Yin, Chung-Wah Siu

**Affiliations:** Department of Echocardiography & Non-invasive Cardiology Laboratory, Sichuan Academy of Medical Sciences & Sichuan Provincial People’s Hospital, Chengdu, China; Division of Cardiology, Department of Medicine, The University of Hong Kong, Hong Kong Special Administrative Region; Department of Echocardiography & Non-invasive Cardiology Laboratory, Sichuan Academy of Medical Sciences & Sichuan Provincial People’s Hospital, Chengdu, China; Division of Cardiology, Department of Medicine, The University of Hong Kong, Hong Kong Special Administrative Region

Dear Editor,

Racial disparity in prevalence and incidence of atrial fibrillation (AF) are known to exist [1], [2]. Specifically, the reported prevalence of AF is around 2% in adult Caucasian population but range from 0.1% to 1.1% in Asian Countries and regions [1]. Paradoxically, many traditional risk factors for AF including hypertension are in fact more prevalent in Asians than Caucasians. In a recently published article in the *Journal* [3], Suwanwela and colleagues reported the results of a community-based AF screening program involving 13,864 residents aged 65 years or above from 11 districts in Lopburi and Phetchaburi province, Thailand. The authors should be congratulated for their success in performing this highly labor-intensive important epidemiological study. The overall prevalence of AF is 2.8%, which increases progressively with age from 2.17% in those aged 65–74 years to 3.9% in those aged ≥75 years. While such prevalence remains lower than that from primarily Caucasian population (~6.13%) in the ATRIA study [4], the prevalence of AF alarmingly increases by nearly 5-fold over the past 2 decades in Thailand [3], [5], [6] (Fig. 1).

**Fig. 1:**
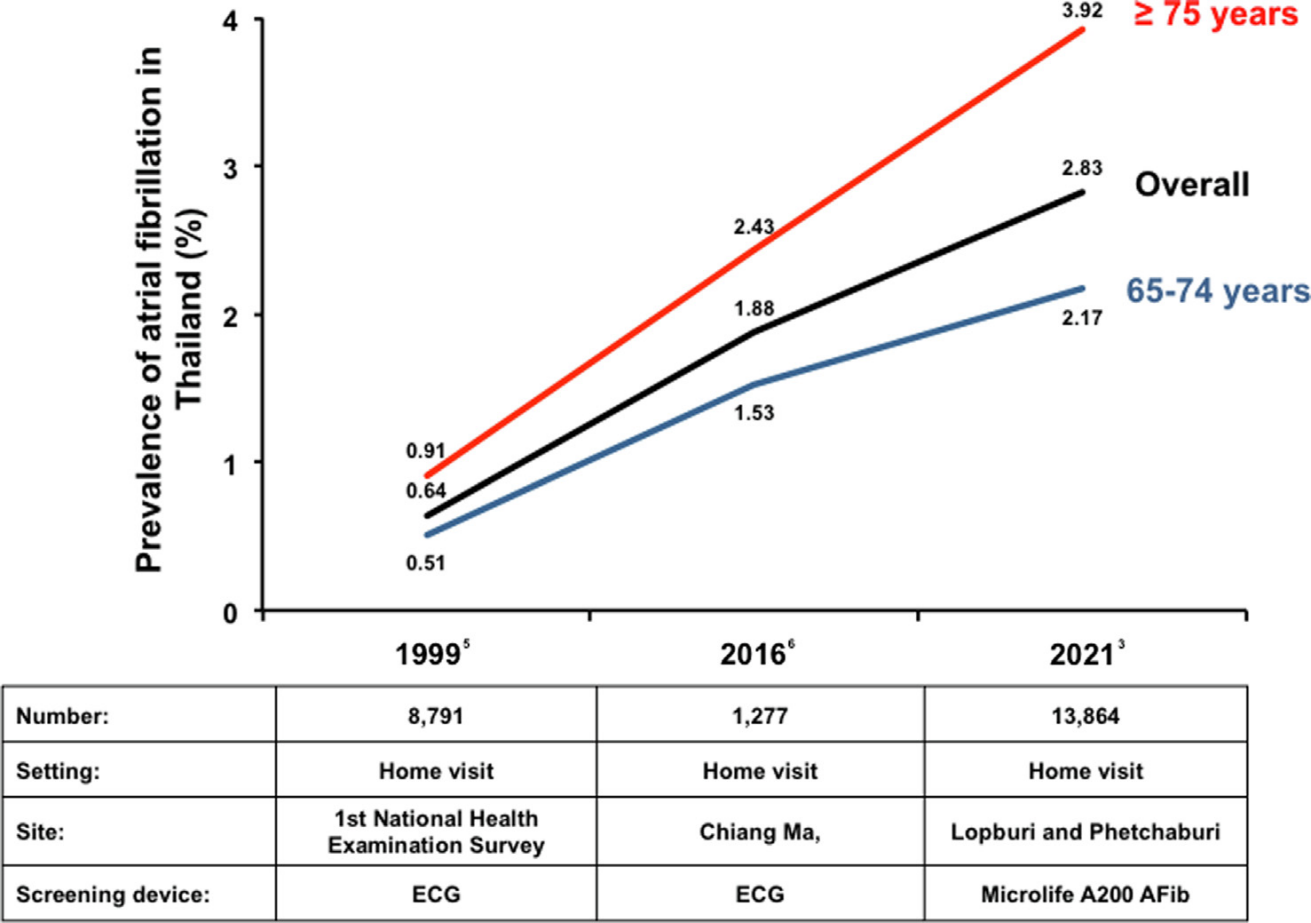
Secular trends of prevalence of atrial fibrillation in Thailand.

While the reported increase in AF prevalence in Thailand is in concordance with the previous projection in Asian countries [7], there are important differences in AF screening strategy, which may confound the results. In contrast to the 2 previous community AF screening studies in Thailand in 1999 and 2016 [5], [6], in which ECG was used for AF detection, trained nurses and village health volunteers performed AF screening using a blood pressure device with AF algorithm (Microlife A200 AFib) during home visits in the report by Suwanwela and colleagues [3]. Participants with possible AF were given follow-up appointment for further investigations including 12-lead ECG and echocardiogram. In fact, our team has previously reported the use of similar the blood pressure device (Microlife WatchBP Home A) for AF screening in a primary care setting with simultaneous single-lead ECG to ascertain the diagnosis of AF [8], [9]. Despite the very high specificity (98.7%) and negative predictive value (99.8%), the relatively low sensitivity of 80.6% of the device results in a high false-positive rate (~50%) [8], [9]. Nonetheless, the device remains an optimal choice for the initial screening in the 2-stage AF screening strategy, provided that subsequent standard 12-lead ECG could be performed for those screened positive with Microlife A200 AFib for an accurate arrhythmia diagnosis. Unfortunately, only 58% of participants detected to have AF using Microlife A200 AFib in the Thai study [3] underwent subsequent ECG, in whom only 33% were confirmed to have AF. The overall prevalence of true AF would be overestimated.

Notwithstanding, epidemiological data of AF in Asian Pacific region is scare, high quality data similar to that from Suwanwela and colleagues is urgently needed to inform policymakers and clinicians in the allocation of limited health care resources.

## Declaration of Competing Interest

The authors declare that they have no known competing financial interests or personal relationships that could have appeared to influence the work reported in this paper.
